# A path to sustainable and healthy diets: modeling ovo-lacto-vegetarian food-based dietary guidelines

**DOI:** 10.3389/fnut.2026.1754132

**Published:** 2026-06-24

**Authors:** Lisa Sturm, Lena Klausmann, Katrin Seper-Nagl, Oliver Alber, Antonia Griesbacher, Tilman Kühn, Klemens Fuchs, Alexandra Wolf, Karl-Heinz Wagner

**Affiliations:** 1 Department Center for Nutrition and Prevention, Austrian Agency for Health and Food Safety, Division Integrative Risk Assessment, Data and Statistics, Vienna, Austria; 2 Vienna Doctoral School of Pharmaceutical, Nutritional and Sport Sciences (PhaNuSpo), Vienna, Austria; 3 Department Statistics and Analytical Epidemiology, Austrian Agency for Health and Food Safety, Division Integrative Risk Assessment, Data and Statistics, Vienna, Austria; 4 Department of Nutritional Sciences, University of Vienna, Vienna, Austria; 5 Department of Nutritional, Food and Consumer Sciences, Fulda University of Applied Sciences, Fulda, Germany; 6 Medical University of Vienna, Center for Public Health, Vienna, Austria; 7 Co-Center for Sustainable Food Systems and Institute for Global Food Security, Queen's University Belfast, Belfast, United Kingdom; 8 Austrian Agency for Health and Food Safety, Division Integrative Risk Assessment, Data and Statistics, Vienna, Austria

**Keywords:** food-based dietary guidelines, healthy diets, mathematical optimization, sustainable diets, vegetarian diets

## Abstract

**Introduction:**

The development of healthy and sustainable food-based dietary guidelines (FBDGs) is an essential measure to support the transformation to sustainable and resilient food systems. Shifting to more sustainable and healthy plant-based diets can benefit both human and planetary health, provided these diets are nutritionally adequate, healthy, environmentally friendly, and culturally acceptable.

**Methods:**

This study aimed to obtain a nutritionally adequate and healthy ovo-lacto-vegetarian diet that reduces greenhouse gas emission (GHGE) and land use (LU) by over 45% in comparison to the average observed diet among Austrian adults, while having the least deviation possible in terms of composition from this average observed diet using mathematical optimization. The results of the optimized diet served as a basis for deriving the ovo-lacto-vegetarian FBDGs.

**Results:**

The optimized diet met all dietary reference values (DRVs) except for docosahexaenoic acid (DHA) and eicosapentaenoic acid (EPA) and was associated with a potential 41% reduction in disability-adjusted life years (DALYs) compared to the observed diet-related burden of disease in the Austrian population. It resulted in GHGE of 2.5 kg CO_2_–eq (carbon dioxide equivalent), and a LU of 2.9 m^2^/day, corresponding to reductions of 58% and 61%, respectively. The share of energy from animal-based foods decreased from 29 E% (percentage of daily energy intake) in the observed diet to 21 E% in the optimized diet. Plant-based foods provided 65% of total protein in the optimized diet. The derived ovo-lacto-vegetarian FBDGs emphasize vegetables, fruits, grains and legumes, with only small amounts of animal-based foods, and recommend higher intakes of legumes and plant-based meat alternatives to support a shift toward a more sustainable diet.

**Discussion:**

This study demonstrates that it is possible to model ovo-lacto-vegetarian FBDGs that are healthy, nutritionally adequate and environmentally sustainable, offering a promising approach for future dietary guidelines. National-level dietary modeling should form the basis for the development of healthy and sustainable FBDGs in the future.

## 1 Introduction

Dietary changes are urgently needed to reduce the high burden of diet-related diseases and to establish sustainable and resilient food systems for a growing global population (1–4). Western diets consisting of high levels of sugar, saturated fat, salt and red and processed meat, are linked to chronic non-communicable diseases (NCDs). In contrast, pre-dominantly plant-based diets are well-known for their health benefits (1, 2, 5, 6). Worldwide, a total of 11 million deaths [out of 55.9 million deaths in total (7)] and 255 million disability-adjusted life-years (DALYs) are attributable to dietary factors (1). Food systems that have facilitated unhealthy diets are also a key driver of climate change and environmental crises, with substantial effects on most planetary boundaries (8, 9). For example, food systems are responsible for about one third (21 to 37%) of all greenhouse gas emissions (GHGE) worldwide (10), underscoring the need to incorporate environmental sustainability into food-based dietary guidelines (FBDGs) as a key lever for transforming food systems (3, 11).

FBDGs are evidence-based dietary recommendations designed to ensure adequate nutrient intake and prevent chronic diseases. They serve as the foundation for nutrition and health policies and are crucial for promoting healthy eating habits and lifestyles (12–15). National dietary guidelines are useful tools for creating diets that are healthier and more sustainable. FAO and WHO (16) emphasize the need to shift to more plant-based diets for health and environmental reasons. FBDGs can promote this transition (11). While global scientific targets for healthy diets and sustainable food production have been established by the EAT-Lancet Commission in 2019 (2) and updated in 2025 (17), national FBDGs are essential to account for local and cultural considerations, dietary preferences, and specific health challenges of the respective population. With the increasing popularity of vegetarian diets (18), it is crucial to develop FBDGs that support nutritionally adequate, healthy, and environmentally friendly food choices for this population group. However, most existing national FBDGs provide little or no quantitative guidance specifically tailored to vegetarian populations (19), leaving a growing segment of consumers without evidence-based recommendations. Dedicated vegetarian FBDGs are therefore critical for ensuring nutrient adequacy, addressing specific nutritional vulnerabilities, and supporting informed dietary choices. Given that vegetarian and vegan diets consistently show substantially lower environmental impacts than meat-based diets, adopting plant-based diets represents an important lever for mitigating climate change (20).

To address the complex task of simultaneously evaluating several key aspects of a sustainable diet, i.e. nutrient adequacy, disease prevention, acceptability, and sustainability aspects, the use of mathematical optimization has proven a useful method in the last few years (21–23). In this study, we therefore used mathematical optimization to model a nutritionally adequate, healthy and low-GHGE and low-land use (LU) ovo-lacto-vegetarian diet, forming the basis for the new Austrian vegetarian FBDGs. To the best of our knowledge, we are globally the first to model national vegetarian FBDGs.

## 2 Materials and methods

The model developed in this study takes the form of a general constrained optimization problem and is based on the model of the German Nutrition Society (DGE) (24–26). An optimization model consists of decision variables, constraints, and an objective function. The optimization method, in particular linear programming, characterizes the solutions of a constrained optimization problem and tries to find the value that each variable must have to comply with the constraints while optimizing (i.e., minimizing or maximizing) the specific value given by the objective function. In diet optimization models, the decision variables represent the quantities of foods to be optimized. The solutions to the constrained optimization problem identify the quantity of each food in the optimized diet that fulfills all constraints for the minimum (or maximum) value of the objective function simultaneously (22, 27).

### 2.1 Input data used for diet modeling

Dietary intake data of the Austrian Nutrition Report 2017 (28) from the EFSA Comprehensive Database (29, 30) was used for diet optimization in the present study. For the Austrian Nutrition Report 2017 a nationally representative sample of the Austrian adult population aged 18–64 years was obtained using a multistage stratified cluster sampling design. Data on food consumption was collected by using a repeated 2 × 24 h recall. Additionally, a 28-item food propensity questionnaire and a socio-demographic questionnaire were used to gather information on habitual dietary intake and socio-economic characteristics (31). Due to the small sample size for vegetarian diets, omnivorous dietary intake data were used. We retrieved dietary intake data from 2.129 adults aged 18–64 years (63% women, 37% men), which are coded using the EFSA's FoodEx2 food classification system (32). The FoodEx2 classification system consists of 21 top-level food groups each of which is further split into up to seven levels of food subgroups and/or foods. FoodEx2 facets, which further describe attributes of a food group, were not considered in this study. As the FoodEx2 food groups do not always correspond to the food groups of FBDGs, some adaptations had to be made in the food grouping of whole grain and other grain products as well as processed meat as described in Schäfer et al. (26). In the present study, some additional adaptations were made e.g. to the grouping of legumes to make it more appropriate for designing FBDGs. For example, pulses such as chickpeas, peas, lentils etc. were allocated to the food group “Legumes, nuts, oilseeds and spices” at FoodEx Level 1 instead of the food group “Vegetables and vegetable products”. For the optimization model the FoodEx2 codes were used at level 5, as this provides the needed level of detail.

The number of food groups, and therefore decision variables, was 662 at level 5. Those food groups were further aggregated into 20 main food groups (Supplementary Table 1) for the reporting of results and further condensed to eight FBDG-food groups for presentation as FBDGs. So-called discretionary foods, such as sweets, sugar-sweetened beverages etc., were reported as the summary of their energy share in addition to the 20 food groups. Discretionary food groups and their respective FoodEx2 food groups are displayed in Supplementary Table 2.

For each decision variable, there is a dataset that includes information on nutrient composition, environmental impact, observed dietary intake, and diet-health relationships. Data on the nutritional content of foods was obtained from the German Food Code and Nutrient Data Base (Bundeslebensmittelschlüssel (BLS) version 3.02) (33) and the Austrian Food Composition Database [Österreichische Nährwerttabelle (ÖNWT)] (34), which include detailed information on macro- and micronutrients. The nutrient content of composite dishes is calculated in the BLS by combining the nutrient data for the individual ingredients. This standardized approach accounts for changes in weight and nutrient content resulting from cooking, frying, and other preparation processes (35).

Environmental data, including greenhouse gas emissions and land use, were sourced from the SHARP indicator database (36). The SHARP database was selected since it provides harmonized, life cycle–based estimates of environmental impacts (integrating production, transport, and consumption-stage adjustments) for a wide range of foods. Furthermore, it was developed to be applicable to European dietary datasets, enabling linkage with individual food consumption data via FoodEx2.

Data on the health impacts DALYs of foods on the Austrian population were derived from the Global Burden of Disease Study (1) and Schwingshackl et al. (37).

### 2.2 Data storage and handling

For ease of use, further development, and data storage, the input data is stored in a MariaDB database and all data interactions are handled with the R programming language version 4.1.3 (38) and its MariaDB database package RMariaDB version 1.2.2 (39). This allows easy and efficient access to the data for the optimization routine and for extending and adapting the data. Two main data bases have been set up. A productive environment for the final data, and a development environment for testing new data in combination with the optimization routine.

### 2.3 Constraints

Acceptability constraints were applied to ensure modeled consumptions per food group remained within the range observed in the target population. Upper limits for the intake were set at the 95th percentile of the observed intake for all individuals for each food group at level 1 and additionally, for each food group on level 5, the 95th percentile for consumers only. For “composite dishes” and “seasoning and sauces,” which are part of the discretionary food groups, a different approach as described by Schäfer et al. (26) was applied, as sensitivity analyses indicated that the model would otherwise assign unrealistically high quantities to these categories. Therefore, based on the lower energy target of the optimized diet compared to the observed diet, the upper acceptability constraints derived from observed intakes were scaled to align with the optimized diet's energy goal. For fruit juices a maximum amount of 80 ml/day was set as a constraint based on the mean consumed amount in the observed diet, because the sensitivity analyses showed that, without additional constraints, the model tended to use fruit juice as a disproportionately high contributor to meeting certain nutrient requirements. This would not align with practical dietary recommendations, which limit fruit juice consumption. We therefore introduced an upper limit based on the observed intake. Additional constraints on total quantity of food, total solid quantity of food and total energy intake were also applied. The equations of the constraints are presented in Supplementary Table 3.

### 2.4 Objective function and nutrient goals

The objective function of the constrained diet optimization problem aims to a) minimize deviation from observed dietary intake, b) minimize environmental impact and c) minimize burden of disease (DALYs). Additionally, nutrient goals must be fulfilled. The optimization problem and the objective function *f* can be expressed as


$$\begin{array}{l} minimize_{x^{opt}}\;f=\left[\begin{array}{c} W^{DA}\times deviation\;food\;habits+\\ W^{SUS}\times environmental\;impact+\\ W^{DHR}\times health\;impact \end{array}\right]+P\;\times \;dfnr\\ \;with\;W^{DA}\text{+}W^{SUS}\text{+}W^{DHR}\text{= 1,}\\ deviation\;food\;habits=\frac{\sum_{i=1}^{n}||\left(x_{i}^{opt}-x_{i}^{obs}\right)||}{Total\;obs\;amount}or\frac{\overset{n}{\underset{i=1}{\sum }}\frac{||\left(x_{i}^{opt}-x_{i}^{obs}\right)||}{x_{i}^{obs}}}{n},\\ environmental\;impact=\left(\overset{n}{\underset{i=1}{\sum }}\alpha \;\left(\frac{x_{i}^{opt}\times GHGE_{i}}{Total\;obs\;GHGE}\right)+\;\beta \;\left(\frac{x_{i}^{opt}\times LU_{i}}{Total\;obs\;LU}\;\right)\right),\\ health\;impact=\overset{n}{\underset{i=1}{\sum }}\delta \times \frac{x_{i}^{opt}\times {DALY_{GBD}}_{i}}{Total\;obs\;DALYs_{GBD}}+\partial \\ \times \frac{x_{i}^{opt}\times {DALY\text{\_}SCHW}_{i}}{Total\;obs\;DALYs\text{\_}SCHW},\text{ and} \end{array}$$

$$\begin{array}{l} dfnr=\sum_{k=1}^{\begin{array}{c} k=nutrients\\ min \end{array}}Dev_{k}^{-}+\sum_{l=1}^{\begin{array}{c} l=nutrients\\ max \end{array}}Dev_{l}^{+}+\sum_{m=1}^{\begin{array}{c} m=nutrients\\ target \end{array}}\frac{\left\|\left(nut_{m}^{opt}-DRV_{m}^{target}\right)\right\|}{DRV_{m}^{target}}\;with\\ Dev_{l}^{+}=\{\begin{array}{ll} \frac{nut_{l}^{opt}-DRV_{l}^{max}}{DRV_{l}^{max}} & if\;nut_{l}^{opt}\geq DRV_{k}^{max}\\ 0 & if\;nut_{l}^{opt}<DRV_{k}^{max} \end{array}\\ subject\;to\;g_{t},t=1,\;\ldots ,\;5. \end{array}$$

W^DA^, W^SUS^, W^DHR^ are weights in order to favor dimensions over others. *n* is the number of selected food groups. $x_{i}^{opt}$and $x_{i}^{obs}$ are the optimized and selected amounts of food group *i* respectively. GHGE_i_ and Lu_i_ represent carbon impact and land use expressed per gram of selected food group *i*. The weights α and β control the environmental metrics, in particular, if carbon impact or land use should be favored. *Total obs GHGE* and *total obs LU* are the total amounts of GHGE and LU in the observed diet. *DAL*_*Y*_*GBD*_*j*_ *and DAL*_*Y*_*SCHW*_*j*_, respectively, stand for the diet health relationship metrics of the Global Burden of Disease Study (GBD) (1) and Schwingshackl et al. (37), expressed per gram of the selected food group *j*. The weights δ and ∂ control the health impact or DALY metrics, in particular if GBD or Schwingshackl should be favored. Total obs *DALYs*_*GBD*_ and total obs *DALYs*_*Schw*_ are the total amounts of GBD and Schwingshackl metrics of the observed diet. *P* is the nutritional deviation tolerance weight. $Dev_{k}^{-}$ is inadequate intake (negative deviations) and $Dev_{l}^{+}\;$is the excess (positive deviations). *k*, *l*, and *m* are numbers of nutrients having a minimum, maximum and target goal respectively. $nut_{k}^{opt}$is the optimized amount of nutrient *k*. $DRV_{k}^{\min}\;$is the minimum DRV (dietary reference value) associated with nutrient *j*. $DRV_{l}^{\max}$ is the maximum DRV associated with nutrient *l*, and $DRV_{m}^{target}$ is the exact target value to reach for nutrient m.

The additional component *dfnr* (*deviation from nutritional requirements)* aims to minimize deviations from minimal nutritional values to reach, to minimize excess for nutrients having a maximal value, and to minimize deviations from nutritional targets. A high value for weight P was assigned to effectively penalize the objective function value if the solution deviates from nutrient goals.

The terms *g*_1_, *g*_2_, *g*_3_, *g*_4_, and *g*_5_ refer to particular inequality and equality constraints for each selected food group, food group level, total quantity, total solid quantity, and total energy intake, respectively. The specific form of these constraints can be found in Supplementary Table 3.

The nutrient goals (Supplementary Table 4) were based on the DRVs for Austria (40) and complemented by EFSA's tolerable upper intake levels for nutrients (41). DRVs for adults [18–65 years, normal weight with moderate physical activity level (PAL 1.4)] were taken and weighted according to the age and sex-specific proportion of the Austrian population. Nutrients such as iodine, selenium, copper, fluoride and manganese were not included due to limited data quality in the nutrient databases used (33, 34). No lower limit was set for vitamin D, as the reference value cannot be achieved solely through dietary intake (42).

Agronomic dependencies for milk and butter were introduced to take into account that in the production of dairy products, butter is produced in a ratio of 5 g butter per 100 g milk. If half of the milk consumed is whole milk, 2.5 g of butter per 100 g of milk must be assumed. The model must therefore comply with the ratio of milk to butter (100:2.5–5) (43). Agronomic dependencies are considered as a nutrient in the objective function.

The optimization, in particular the linear and quadratic programming routine, was implemented with the R programming language and the package ROI 1.0.1 (44) and its two plugins ROI.plugin.lpsolve 1.0.1 (45), and ROI.plugin.quadprog 1.0.1 (46) for linear programming. It has to be noted that linear programming was used as the default method.

For practical use, particularly for testing different values in the constraint equations or additional intake data, the optimization was made available within an interactive web application, which was developed with R and the web application package shiny version 1.7.4 (47). Test and production applications are connected to the intake data bases mentioned in section 2.2. The optimization can thus access the data directly and be rerun in case of new intake data or constraint settings.

### 2.5 Analyses

In order to assess the sensitivity and robustness of the model results, different weightings for the three components (deviation from observed dietary intake, environmental impact, health impact) of the objective function were tested. GHGE and LU, as well as DALYs from GBD and Schwingshackl, were equally weighted within their component of the objective function. Our analyses showed that a stronger consideration of the health and environmental components led to similar results, whereas a higher weighting of the “deviation from observed dietary intake” deviated from this. A step-by-step approach was then used to assess a weighting for the “deviation from observed dietary intake” parameter, that yields best possible results in the optimization in section 2.4 and at the same time also adheres to practical and realistic dietary patterns. The weighting of this parameter was tested in an interval [0, 100] in steps of 5, while the weighting of the other two parameters remained equally distributed. The present study also aimed to reduce the environmental impact of the resulting dietary patterns by at least 45% according to IPCC (48), as a 45% reduction in greenhouse gas emissions by 2030 could limit global warming to 1.5 °C with a 50% probability (49). The step-by-step procedure mentioned above resulted in a weighting factor of 30% for the “deviation from observed diet” parameter, and 35% for environmental impact and health respectively and therefore achieves the environmental target.

### 2.6 Derivation of ovo-lacto-vegetarian FBDGs for Austria

In order to provide consistent and understandable messages to consumers, the results of the optimization calculations were converted from grams into recommended daily or weekly portions in practical serving sizes. This process was carried out iteratively over several meetings with a wide range of (inter)national experts and over 40 stakeholders of the National Nutrition Commission of the Federal Ministry of Social Affairs, Health, Care and Consumer Protection.

### 2.7 Calculation of the health impact of the FBDGs

The DALYs of the optimized diet were calculated as follows:


$$\begin{array}{r} DALYs\;per\;100\;g\;of\;food\;group\;i=\;\frac{\left(DALYs_{i}^{Obs}-0\;DALYs\right)}{\left(x_{i}^{Obs}-x_{i}^{TMREL}\right)}\times 100\\ DALYs_{opt}=\sum_{x_{i}}\frac{DALYs\;per\;100\;g\;of\;i\;\times \;x_{i}^{opt}}{100} \end{array}$$

The underlying assumptions for establishing an indicator for diet-health relations can be found elsewhere (25).

## 3 Results

### 3.1 Results of optimization calculations—Food composition

The amounts of foods in the observed and optimized diet are shown in Table 1. Overall, the ratio of plant-based foods to animal-based foods is higher in the optimized diet (68:32) compared to the observed diet (55:45). In particular, the optimized diet contains higher amounts of vegetables, potatoes, plant-based meat alternatives, plant-based drinks, eggs, vegetable fats and spreadable animal-based fats. No major differences are found regarding fruits, legumes, whole grains as well as nuts and seeds. A high amount of milk and dairy products is preserved in the optimized diet. Moreover, the optimized diet contains substantially less discretionary foods.

**Table 1:** Content of main food groups (g) of the observed diet and the optimized diet.

Food groups and subgroups	Observed diet (g/d)^*^	Optimized diet (g/d)
Drinking water	1,626	790
Coffee and tea	532	61
Vegetables and fruit	394	586
Vegetables	183	375
Fruit	131	131
Fruit and vegetable juices	80	80
Grains, grain-based products and potatoes	301	431
Refined grains (-products)	229	232
Whole grains (-products)	17	17
Potatoes	55	182
Legumes and legume products	14	54
Legumes (cooked)	12	12
Plant-based meat substitutes made of legumes	2	42
Eggs	23	38
Milk equivalents^**^	571	579
Milk/dairy (in milk equivalents)	561	506
Plant-based drinks	9	73
Fats, oils, nuts and seeds	25	32
Vegetable oils	8	12
Nuts and seeds	8	7
Spreadable fats	9	13
Meat and fish	147	—
Red meat	56	—
Processed meat	44	—
Poultry	28	—
Fish and seafood	19	—
Discretionary foods	21 E%	7 E%

^*^Omnivorous consumption data (28) was used as reference data for the model, as the sample was too small for vegetarian diets.

^**^A milk equivalent is a measure used to calculate the amount of milk used in a dairy product. Conversion of dairy products into milk equivalents (115): milk factor 1; Dairy products factor 1.4; Cheese factor 7.2.

Most of the overall energy in both diets shall from grains and milk and dairy products. The proportion of energy from all animal-based foods is 21 E% (percentage of daily energy intake) in the optimized diet and 29 E% in the observed diet, respectively. Discretionary energy decreases from 21 E% in the observed diet to 7 E% in the optimized diet.

In the optimized diet, plant-based foods contribute to 65% of total protein. 35% of protein comes from animal-based foods. Grains (37%) followed by milk and dairy products (28%) represent the largest share. This compares to 44% of total protein in the observed diet coming from plant-based foods and 56% from animal-based foods. In the observed diet however, grains (36%), milk and dairy products (20%) and red meat (14%) are the main contributors of total protein.

### 3.2 Nutritional adequacy of the ovo-lacto-vegetarian diet

The nutrient contribution of the observed and optimized diets are shown in Supplementary Table 5. The observed diet contains suboptimal amounts of energy (2,250 kcal), fat, saturated fat, cholesterol, dietary fiber, free sugars, folate, calcium, iron, potassium and sodium compared to the targeted amounts. The optimized diet, however, is within the acceptable distribution ranges for macronutrients and meets all nutrient goals. The optimized diet provides 2,029 kcal and consists of 53 E% carbohydrates, 30 E% fat and 64 g protein per day. Vitamin B_12_ is by far the most limiting (binding) nutrient and therefore the strongest determinant of the dietary changes required from the observed diet. Vitamin B_12_ content in the optimized diet (4 μg/d) is lower than in the observed diet (6 μg/d) but meets the DRVs. Eggs and milk/dairy are the main sources of B_12_ in the optimized diet and contribute to 51% (milk and dairy) and 17% (eggs and egg products) to the fulfillment of the nutrient goal. In contrast, in the observed diet, the main sources for B_12_ are red meat (30% of total amount of B_12_) and milk and dairy (26% of total amount of B_12_). Iron content shall be 15 mg/d in the optimized diet and 12 mg/d in the observed diet, respectively. In the optimized diet, refined cereal products (31% of total iron) and vegetables (30% of total iron) contribute the most of the iron supply. The DRVs for the essential fatty acids linolic acid (LA; omega-6) and α-linolenic acid (ALA) (omega-3), which are the precursors of arachidonic acid (ARA; omega-6), eicosapentaenoic acid (EPA, omega-3) and docosahexaenoic acid (DHA, omega-3) in humans, are met. Due to the lack of fish and seafood in an ovo-lacto-vegetarian diet, no nutrient goals were set for DHA and EPA, and additional supplementation was no option for the general recommendation. Therefore, DHA and EPA content of the optimized diet was relatively low with 47 mg/d, coming primarily from eggs (60% of total DHA/EPA).

### 3.3 Environmental and health impact

The GHGE of the observed diet is 5.9 kg CO_2_-eq (carbon dioxide equivalent) and LU is 7.3 m^2^/year/day (Table 2). The GHGE of the optimized diet is 2.5 kg CO_2_-eq, LU is 2.9 m^2^, respectively. Shifting to a vegetarian dietary pattern is estimated to lead to reductions in GHGE (58%) and LU (61%). In the observed diet, animal-based foods contribute the most to GHGE (61%). This is pre-dominantly from red meat (23%), which is also the largest contributor to impacts on LU (32%). In the optimized diet, the main contribution to GHGE as well as LU are milk and dairy products (34%). The optimized diet would result in a potential reduction of 41% DALYs compared to the observed diet-related burden of disease in the Austrian population (Table 2). For details see Supplementary Table 6. An even greater reduction could be achieved by increasing the proportion of whole grains in one's diet.

**Table 2:** Overview of GHGE, LU and DALYs of the observed and optimized diet.

Environmental and health indicators	Observed diet	Optimized diet
GHGE (kg CO_2_-eq/day)	5.86	2.49
Land use (m^2^/year/day)	7.34	2.86
Reduction in DALYs	—	41%

### 3.4 Recommended daily amounts for food groups—Derivation of FBDGs

The results of the optimized dietary pattern served as a basis for deriving the ovo-lacto-vegetarian FBDGs. The optimized dietary pattern in grams were converted into recommended daily or weekly servings in practical serving sizes. For this purpose, standard portion sizes were defined and the corresponding number of portions was calculated. For example, one portion of eggs was set at 60 g; thus, a consumption of 38 g/day corresponds to 266 g per week, i.e. approximately four eggs. Similarly, one portion of fruits or vegetables was defined as 110 g; therefore, an intake of 586 g/day equals about 5.3 portions per day. Table 3 shows the translation into ovo-lacto-vegetarian FBDGs for Austria. Plant-based protein sources, particularly legumes and minimally processed plant-based meat alternatives (i.e. tofu, tempeh or textured soy protein), constitute a central component of the recommendations, with an intake of at least four weekly servings. Achieving the DRV for vitamin B_12_ required three daily servings of milk and dairy products and four eggs per week in the optimized pattern. Fortified plant-based drinks were also included in the modeling; however, given their heterogeneous nutrient profiles and limited comparability with cow's milk, they are referenced only in the supplementary material of the FBDGs (53). People who consume less milk and dairy products should opt for fortified, unsweetened plant-based products (e.g. soy drinks) that are fortified with calcium, vitamin B_12_ and vitamin B_2_.

**Table 3:** National ovo-lacto-vegetarian FBDGs of Austria.

Food group	Recommended daily or weekly portions	Portion size
Non-alcoholic beverages (no added sugars or sweeteners)	Six portions/day	250 ml
Vegetables and fruit	Five portions/day	Vegetables, cooked: 200–300 g Vegetables, raw: 100–200 g Salad: 75–100 g Fruit: 125–150 g
Grains and potatoes	Five portions/day	Bread: 50–70 g Rolls: 50–70 g Cereals: 50–60 g Pasta, uncooked: 65–80 g Rice, cooked: 150–180 g Potatoes: 200–250 g
Legumes and legume products	At least four portions/week	Legumes, cooked: 125 g Tofu, Tempeh etc.: 80 g
Milk and dairy	Three portions/day	Milk: 150–200 ml Yogurt: 150–200 g Cheese: 50–60 g
Eggs	Four portions/week	60 g (one egg)
Vegetable oils, nuts and seeds	Two portions/day	Vegetable oil: one tablespoon (10 g) Nuts and seeds: two tablespoons (25 g)
Foods high in fat, sugar and salt	Occasionally	

## 4 Discussion

The present paper describes the process and results of the development of the first ovo-lacto-vegetarian FBDGs for Austria. In line with other data (21, 22, 50, 51), we showed that mathematical optimization is a suitable tool to formulate FBDGs as it offers a holistic approach to manage several dimensions of a healthy and sustainable diet at the same time. In the last few years, this innovative approach has been successfully applied in the context of sustainable FBDGs development in countries such as the Netherlands, Germany and Switzerland (21, 24, 52). FBDGs from these countries have in common that they emphasize plant foods, with a smaller proportion of animal-based foods. In Austria, we are the first to issue official national ovo-lacto-vegetarian FBDGs that were explicitly derived using mathematical optimization, complementing our previously modeled FBDGs for omnivorous adults (53). Currently, around 11% of the Austrian population are vegetarians (54), which underlines the relevance of our specific recommendations for this growing population (55).

### 4.1 Environmental impact

Consistent with our findings, modeled dietary scenarios have demonstrated that vegetarian and vegan diets substantially reduce environmental impact compared to meat-based diets (20, 56, 57). Our findings align with extensive data indicating that the consumption of animal products, particularly meat and sausages, is linked to very high GHGE (57–59). In the present study, shifting to a more plant-based vegetarian diet is estimated to lead to reductions in GHGE (-58%) and LU (-61%), in comparison to the observed diet of the Austrian population, which is in line with findings from Bunge et al. (60) that showed that shifting to vegan and vegetarian diets has the largest environmental impact reduction potential compared to other diets. Vegetarian and especially vegan diets have consistently been shown to cause the least climate impact in modeling studies (61). This pattern is also observed for other indicators such as water use (20, 62). However, when considering water in more detail, some plant-based foods, such as nuts (e.g., almonds and cashews), are major contributors to the blue water footprint (i.e., irrigation) (63, 64). Thus, by offering dietary recommendations that encompass vegetarian diets with lower amounts of animal-based products, FBDGs can significantly contribute to ecological sustainability (65).

### 4.2 Health impact

Our findings align with data indicating that FBDGs recommending plant-based diets as a way to prevent and control NCDs are strongly supported by their proven beneficial effects, including the protection against pre-mature mortality (66–70). A recent modeling study further supports the association between vegetarian diets and reduced pre-mature mortality (71). Compared with omnivorous diets, vegetarian dietary patterns are associated with lower total cholesterol and LDL cholesterol levels and reduced body weight. In addition, individuals following vegetarian diets exhibit a significantly decreased risk of type 2 diabetes, ischemic heart disease, and selected cancers (72). Eating vegetarian diets rich in minimally processed plant foods has been linked to a reduced risk of developing various chronic diseases, such as diabetes, hypertension, cancer (73), cardiovascular disease and ischemic heart disease (74). A diet that is primarily plant-based and low in added sugars, saturated fats, and salt contributes to a healthy lifestyle and is associated with a lower risk of pre-mature mortality and protects against NCDs (75). Reducing meat and animal protein intake e.g. in high-income countries, where consumption exceeds recommended levels and protein intake surpasses requirements, can yield health benefits and lessen the environmental impact of food consumption (76). However, since the early 1960s, the meat intake has meanwhile increased in several transition countries such as China or Brazil (77) and a return to a more plant-based diet would also lower the NCD rate in many middle income countries (78). In addition to the individual health benefits, adopting plant-based diets could save billions of euros in healthcare costs across Europe but also globally. Excessive meat consumption challenge poses a challenge to global healthcare systems; for instance, in 2020, it was estimated that 2.4 million deaths worldwide and around €240 billion in healthcare expenses were attributable to excessive red and processed meat consumption (75, 79).

### 4.3 Nutritional adequacy

Some nutrients may be critical in ovo-lacto-vegetarian diets, particularly iron, vitamin B_12_, DHA/EPA, iodine and protein (80–82). In the present work, we modeled a nutritionally adequate ovo-lacto-vegetarian diet for all nutrients, with the exception of EPA/DHA, because fish and seafood are not part of an ovo-lacto-vegetarian diet. However, plant-derived ALA can be converted into EPA and DHA, especially when the consumption of LA is adequate (83, 84). Therefore, only LA and ALA are essential fatty acids in the recommendations of the German Speaking countries (40) and their intake is met with the new ovo-lacto vegetarian guidelines. Data on EPA and DHA also show that vegetarians do have lower levels of EPA and DHA in their plasma, serum, erythrocytes, adipose tissue, and platelets compared to omnivores (83), but there is no evidence that this impacts heart health or cognitive function negatively (81, 85). Vegans and vegetarians may have a more efficient conversion from ALA to EPA and DHA (86). Currently the recommendations for the German speaking countries have not established recommendations for EPA and DHA, while the European Food Safety Authority recommends an intake of 250 mg/day for EPA and DHA (87). Therefore, an increased intake of ALA rich foods i.e. canola oil, flaxseed oil, hemp seed, walnuts and chia seeds and a moderate intake in LA rich foods such as sunflower oil, safflower oil, corn oil, soya oil, peanuts and avocados (33) is, especially in vegetarian and vegan diets, recommended to meet an optimal n-3 to n-6 ratio (83). Nevertheless, we acknowledge that further research is needed on the conversion of ALA to EPA under real-world conditions. Microalgae oils and foods enriched with them are a plant-based source of EPA and DHA (88).

DRVs for vitamin B_12_ were achieved by recommending three portions of milk and dairy products daily and four eggs per week. Data from the EPIC-Oxford study show that 7% of vegetarians have concentrations below 118 pmol/l indicating B_12_-deficiency, mainly due to a lower B_12_-intake than meat-eaters (89). It is therefore essential in communication of FBDGs to inform consumers about the importance of adequate food intake, particularly for vitamin B_12_.

The recommendations for iron were also achieved. People following an ovo-lacto-vegetarian diet can achieve a comparable or even higher iron intake than people with an omnivorous diet (90). However, due to the lower bioavailability of non-haeme iron from plant foods, studies have shown that ovo-lacto-vegetarians may have lower serum ferritin levels and a higher risk of iron deficiency, including iron deficiency anemia (91, 92).

Following a vegetarian or vegan diet may increase the risk of iodine deficiency (82, 93). Plant foods such as fruits, vegetables, and cereals generally provide only limited amounts of iodine because their iodine content depends on the iodine concentration of the soil in which they are cultivated (94, 95). Certain types of seaweed contain very high levels of iodine due to the accumulation of iodine from seawater and may even lead to excessive intake (96, 97). The consumption of iodized table salt and foods produced with it remains an important strategy to ensure adequate iodine intake. In addition, milk, dairy products and eggs are relevant dietary iodine sources due to iodine fortification of feed (82), making them an important component of the new ovo-lacto-vegetarian FBDGs. Iodine intake may be insufficient in diets that exclude dairy products and rely on plant-based substitutes instead (82). In Austria, iodine is added to table salt since 1963 to improve the iodine intake of the population (98).

In terms of protein intake, it is known that different protein sources with complementary indispensable amino acid profiles can be combined in meals to improve the quality of dietary protein in vegetarian diets (99, 100). By combining vegetable protein sources such as pulses, soy products, whole grains, nuts, and seeds with milk, dairy or eggs, a sufficient high-quality protein intake can be achieved (55). In accordance with our results, a recent study showed that the optimal ratio of animal-based proteins to plant-based proteins is about 40:60 (101). Current trends in consumer behavior show a growing demand for plant-based meat alternatives and plant-based drinks. In Austria, the sales of plant-based foods grew by 22% between 2020 and 2022 (102). Consuming unsweetened fortified plant-based drinks (i.e. soy drinks) in addition to dairy products can help to increase diet's sustainability (103). Replacing animal-based foods with plant-based alternatives can reduce the environmental impact of the diet (60). Therefore, they have the potential to support the transition toward more plant-based diets. However, several factors, including the raw materials used, the level of processing, and the geographical origin, should be considered when incorporating them into FBDGs. FBDGs therefore ought to assist consumers in determining which plant-based alternatives to choose (19). However, there is still uncertainty about the long-term health impact of consuming plant-based alternatives (104–106).

To support an adequate intake of potential critical nutrients, the FBDGs therefore include concrete recommendations for these specific nutrients since data have already shown that this is beneficial for consumers (19).

### 4.4 Composition of the vegetarian FBDGs

Healthy and sustainable ovo-lacto-vegetarian FBDGs are high in vegetables, fruits, grains and legumes and contain only small amounts of animal-based foods. The modeled vegetarian FBDGs recommend an increase in the consumption of legumes and plant-based meat alternatives compared to the observed diet of the Austrian population. This is in line with data showing that legumes are a useful tool for facilitating the shift to more sustainable diets (2, 107). Although legumes may not be frequently consumed in many high-income countries, they are a traditional staple in numerous diets. Their consumption is linked to significant health benefits (108), they are affordable, widely accessible, and provide excellent sources of protein, dietary fiber, and micronutrients, while having a low GHGE profile (109).

### 4.5 Strengths and limitations

Our study presents several strengths. First, when modeling diets, we considered apart from EPA and DHA all nutrient reference values. Second, we not only assessed the nutritional adequacy but additionally, we utilized recent and reliable data on DALYs as a proxy to assess the potential health impact of the diets. Third, we also considered cultural acceptability, reflecting the practical feasibility of the solutions. Finally, the inclusion of plant-based meat and milk alternatives was also a major advantage, as this reflects the current consumer behavior of the population. However, our study presents also some limitations. First, we had to use omnivorous dietary intake data as comparison due to the small sample size data available for vegetarian diets of Austrians. Data of the next Austrian Nutrition Report and the ongoing COPLANT-study (https://coplant.univie.ac.at/) could benefit future diet modeling studies. Second, some nutrients (such as iodine, selenium, copper, fluoride and manganese) had to be excluded due to limited data quality in the nutrient databases used. Third, only data on greenhouse gas emissions and land use were used as relevant environmental metrics, as an adequate amount of reliable data is available for these (110). However, GHGE and land use account for the majority of the environmental impact of foods (23). GHGE are by far the most commonly used indicator (111) and also correlate strongly with land use, water use, acidification and freshwater and marine eutrophication (20). Nevertheless, future updates should integrate additional indicators when robust, food-group–level data become available (e.g., biodiversity loss, water scarcity, acidification, and eutrophication), to quantify potential trade-offs more explicitly. Moreover, the DALY estimates rely on simplified, hypothetical assumptions, including a linear dose–response relationship between changes in dietary intake and associated health outcomes. As such, the resulting DALY values should be interpreted as indicative rather than predictive. Finally, affordability was not within the scope of our study. However, previous modeling work suggests that healthy and sustainable dietary patterns, such as vegetarian diets, may reduce food costs in high-income and upper-middle-income countries, while potentially increasing costs in lower-middle-income to low-income settings (116). Furthermore, two recent Austrian studies indicate that the new Austrian ovo-lacto-vegetarian FBDGs may be less expensive compared to the current diet (112, 113). This highlights the importance of considering socio-economic context when translating optimized diets into policy recommendations. One major advantage of the chosen modeling approach is that future improvements regarding databases on nutrients, sustainability indicators or actual dietary intakes in the target population can be incorporated for updated FBDGs.

## 5 Conclusion

To summarize, our study shows that it is possible to obtain nutritionally adequate ovo-lacto-vegetarian FBDGs that lower the burden of disease by 41% for the Austrian population and reduce GHGE by 58% and LU by 61%, in comparison to the observed diet of the Austrian population. This study thus provides an additional argument supporting the shift toward more plant-based diets. In line with other findings (55) we conclude that well-planned, balanced vegetarian diets are nutritious and healthy. Furthermore, adopting plant-based diets could play a crucial role in enhancing the sustainability of our diets (114). Dietary modeling at national level should form the basis for the development of healthy and sustainable FBDGs in the future.

## Data Availability

The original contributions presented in the study are included in the article/supplementary material, further inquiries can be directed to the corresponding author.
