# Supplementary material for: A path to sustainable and healthy diets: modeling ovo-lacto-vegetarian food-based dietary guidelines
Source: Front Nutr. 2026 Jun 24;13:1754132. doi: 10.3389/fnut.2026.1754132 (PMC13341565; doi:10.3389/fnut.2026.1754132)
Supplement: Supplementary file 1 [file Table_1.docx]

Supplement 1: Overview of the 20 main food groups and the according FoodEx2 food group names, codes and levels

| Main food groups | FoodEx2 food group name | FoodEx2 code | FoodEx2  level |
| --- | --- | --- | --- |
| Drinking water | Drinking water | A03DK | 2 |
| Coffee and tea | Coffee, cocoa, tea and infusions | A03GG | 1 |
| Vegetables | Vegetables and vegetable products | A00FJ | 1 |
| Fruit | Fruit and fruit products | A01BS | 1 |
| Fruit and vegetable juices | Fruit and vegetable juices and nectars (including concentrates) | A039K | 1 |
| Refined grains* | Grains and grain-based products | A000J_RG* | 1 |
| Whole grain (-products)** | Grains and grain-based products | A000J_WG** | 1 |
| Potatoes | Potatoes and similar | A0DPP | 3 |
| Legumes (cooked) | Legumes, nuts, oilseeds and spices | A04RG | 1 |
| Plant-based meat substitutes made of legumes | Meat imitates | A03TE | 3 |
| Eggs | Eggs and egg products | A031E | 1 |
| Milk and dairy products | Milk and dairy products | A02LR | 1 |
| Plant-based drinks | Dairy imitates | A0BXC | 3 |
| Vegetable oils | Vegetable fats and oils, edible | A036N | 3 |
| Nuts and seeds | Nuts, oilseeds and oilfruits | A04RH | 2 |
| Spreadable fats | Fat emulsions and blended fats | A039B | 2 |
| Red meat | Mammals meat | A0EYF | 3 |
| Processed meat*** | Processed meat products | A01QR_P*** | 2 |
| Poultry | Birds meat | A0EYG | 3 |
| Fish and seafood | Fish, seafood, amphibians, reptiles and invertebrates | A026T | 1 |

*, **, *** described elsewhere (1)

1. Schäfer AC, Boeing H, Gazan R, Conrad J, Gedrich K, Breidenassel C, et al. A Methodological Framework for Deriving the German Food-Based Dietary Guidelines 2024: Food Groups, Nutrient Goals, and Objective Functions. *PLOS ONE* (2025) 20(3):e0313347. doi: 10.1371/journal.pone.0313347
