# Supplementary material for: A path to sustainable and healthy diets: modeling ovo-lacto-vegetarian food-based dietary guidelines
Source: Front Nutr. 2026 Jun 24;13:1754132. doi: 10.3389/fnut.2026.1754132 (PMC13341565; doi:10.3389/fnut.2026.1754132)
Supplement: Supplementary file 2 [file Table_2.docx]

Supplement 2: Overview of the discretionary food groups and the according FoodEx2 food group names, codes and levels

| Food group | FoodEx2 food group name | FoodEx2 code | FoodEx2  level |
| --- | --- | --- | --- |
| Softdrinks | Water based beverages | A04PY | 2 |
| Alcoholic beverages | Alcoholic beverages | A03LZ | 1 |
| Salty snacks | Snacks other than chips and similar | A06HL | 3 |
| Sweets | Sugar and similar, confectionery and water-based sweet desserts | A032F | 1 |
| Seasoning and sauces | Seasoning, sauces and condiments | A042N | 1 |
| Others | Products for non-standard diets, food imitations and food supplements  FoodEx2 food groups not able to be matched to other groups | A03RQ | 1 |
| Composite dishes | Composite dishes | A03VA | 1 |
