# Supplementary material for: A path to sustainable and healthy diets: modeling ovo-lacto-vegetarian food-based dietary guidelines
Source: Front Nutr. 2026 Jun 24;13:1754132. doi: 10.3389/fnut.2026.1754132 (PMC13341565; doi:10.3389/fnut.2026.1754132)
Supplement: Supplementary file 3 [file Table_3.docx]

**Supplement 3: Equation of constraints**

Acceptability constraints:

For each selected food group (decision variable):

$min{\leq x}_{i}^{opt}\leq max$,

where$x_{i}^{opt}$ is the optimized quantity of selected food group $i$. min = 0 and max = P95 (95% percentile) among consumers only.

For each food group level 1:

$min{\leq G}_{j}^{opt}\leq max$,

where $G_{j}^{opt}$is the optimized quantity of food group level 1 $j$. min = 0 and max = P95 (95% percentile) among all individuals.

Further constraints:

*Total quantity:*

$\min\leq100*\frac{{TOT}^{opt}}{{TOT}^{obs}}\leq max$,

where ${TOT}^{opt}$ is the total optimized quantity and ${TOT}^{obs}$ the total observed quantity. By default, min = 0 and max = $\infty$.

*Total solid quantity:*

$\min\leq100*\frac{{SOL}^{opt}}{{SOL}^{obs}}\leq max$,

where ${SOL}^{opt}$ is the total solid optimized quantity and ${SOL}^{obs}$ the total solid observed quantity. By default, min = 0 and max = 100.

*Total energy intake:*

$\sum_{i=1}^{n} x_{i}^{opt}{*nrj}_{i}={nrj}_{target}$,

where $x_{i}^{opt}$is the optimized amount and ${nrj}_{i}$ = energy content per gram of selected food group $i$. ${nrj}_{target}$ is the total energy content to reach.
