# Supplementary material for: A path to sustainable and healthy diets: modeling ovo-lacto-vegetarian food-based dietary guidelines
Source: Front Nutr. 2026 Jun 24;13:1754132. doi: 10.3389/fnut.2026.1754132 (PMC13341565; doi:10.3389/fnut.2026.1754132)
Supplement: Supplementary file 4 [file Table_4.docx]

Supplement 4: Overview of the nutrient goals

| Nutrient | Type | Lower level | Upper level |
| --- | --- | --- | --- |
| Fat, E% | GV | - | 30 |
| Saturated fatty acids, E% | n.a. | - | 10 |
| Monounsaturated fatty acids, E% | n.a. | 10 | - |
| Polyunsaturated fatty acids, E% | n.a. | 7 | 10 |
| Linoleic acid, E% | RI | 2.5 | - |
| α-Linolenic acid, E% | EST | 0.5 | - |
| Cholesterol (mg/d) | GV | - | 300 |
| Protein (g/d) | RI | 52 | - |
| Carbohydrates, E% | n.a. | - | - |
| Free sugars, E% | n.a. | - | 10* |
| Fiber (g/d) | GV | 30 | - |
| Alcohol (g/d) | GV | - | 10** |
| Vitamin A (Retinol Activity Equivalent µg/d) | RI | 776 | - |
| Vitamin E (Equivalent mg/d) | EST | 13 | 300*** |
| Vitamin K1 (µg/d) | EST | 68 | - |
| Thiamin (mg/d) | RI | 1.1 | - |
| Riboflavin (mg/d) | RI | 1.2 | - |
| Niacin (Equivalent mg/d) | RI | 13.4 | - |
| Pantothenic acid (mg/d) | EST | 5 | - |
| Vitamin B6 (mg/d) | RI | 1.5 | 25*** |
| Biotin (µg/d) | EST | 40 | - |
| Folate (µg/d) | RI | 300 | 1000*** |
| Vitamin B12 (µg/d) | EST | 4 | - |
| Vitamin C (mg/d) | RI | 103 | - |
| Sodium (mg/d) | EST | 1500 | 2400*** |
| Chloride (mg/d) | EST | 2300 | - |
| Potassium (mg/d) | EST | 4000 | - |
| Calcium (mg/d) | RI | 1000 | 2500*** |
| Phosphorus (mg/d) | RI | 700 | - |
| Magnesium (mg/d) | EST | 325 | - |
| Iron (mg/d) | RI | 15** | - |
| Iodine (µg/d) | RI | -**** | 600*** |
| Fluoride (mg/d) | GV | - | 7*** |
| Zinc (mg/d) | RI | 11 | 25*** |
| Copper (mg/d) | EST | - | 5*** |
| Water (ml/d) | GV | 2161 | - |
|  |  | **Equivalent** | |
| Energy, kcal | GV | 2029 |  |

E%: Percentage of daily energy intake, GV = guiding value, RI = recommended intake, EST = estimated value for an adequate intake,

DRVs from DGE/ÖGE (1) unless differently indicated

*Recommendation of the World Health Organization (2)

** Reference value for women instead of weighted average

***European Food Safety Authority Dietary Reference Value/Upper Intake Level (3)

********Exclusion due to data weakness

1. Deutsche Gesellschaft für Ernährung e.V., Österreichische Gesellschaft für Ernährung. *Referenzwerte Für Die Nährstoffzufuhr*. 2 ed. Bonn: Deutsche Gesellschaft für Ernährung und Österreichische Gesellschaft für Ernährung (2024).

2. World Health Organization. Guideline: Sugars Intake for Adults and Children. Geneva: World Health Organization (2015). Available from: <https://iris.who.int/bitstream/handle/10665/149782/9789241549028_eng.pdf?sequence=1>

3. European Food Safety Authority. Overview on Tolerable Upper Intake Levels as Derived by the Scientific Committee on Food and the Efsa Panel on Dietetic Products, Nutrition and Allergies. Parma: EFSA (2024). Available from: <https://www.efsa.europa.eu/sites/default/files/2024-05/ul-summary-report.pdf>
