# Supplementary material for: A path to sustainable and healthy diets: modeling ovo-lacto-vegetarian food-based dietary guidelines
Source: Front Nutr. 2026 Jun 24;13:1754132. doi: 10.3389/fnut.2026.1754132 (PMC13341565; doi:10.3389/fnut.2026.1754132)
Supplement: Supplementary file 5 [file Table_5.docx]

**Supplement 5: Nutrient contents of the observed diet and the optimized diet**

|  | **Observed diet** | **Optimized diet** |
| --- | --- | --- |
| Energy, kcal | 2250 | 2029 |
| Protein, g | 78 | 64 |
| Fat, E% | 37 | 30 |
| Saturated fatty acids, E% | 15 | 10 |
| Polyunsaturated fatty acids, E% | 7 | 7 |
| Monounsaturated fatty acids, E% | 12 | 10 |
| Linoleic acid (LA), E% | 5 | 6 |
| Alpha-linolenic acid (ALA), E% | 1 | 1 |
| EPA+DHA, mg | 377 | 46 |
| Cholesterol, mg | 335 | 300 |
| Carbohydrates, E% | 50 | 53 |
| Free sugars, E% | 13 | 10 |
| Dietary Fibres, g | 22 | 32 |
| Sodium, mg | 2381 | 1500 |
| Chloride, mg | 3744 | 2720 |
| Potassium, mg | 3346 | 4000 |
| Calcium, mg | 959 | 1258 |
| Magnesium, mg | 467 | 442 |
| Phosphorus, mg | 1384 | 1521 |
| Iron, mg | 12 | 15 |
| Iodide, ug | 101 | 92 |
| Fluoride, ug | 1082 | 727 |
| Zinc, mg | 12 | 11 |
| Copper, ug | 1842 | 1950 |
| Manganese, ug | 4700 | 5803 |
| Retinol activity equivalent, ug | 1166 | 3116 |
| Vitamin D-calciferols, ug | 4 | 4 |
| Vitamin E-tocopherol equivalent, ug | 15242 | 28076 |
| Vitamin K, ug | 104 | 454 |
| Vitamin B1, ug | 1562 | 2248 |
| Vitamin B12, ug | 6 | 4 |
| Vitamin B2, ug | 1832 | 2249 |
| Vitamin B3 niacin equivalent, mg | 42 | 30 |
| Vitamin B5, ug | 5445 | 9057 |
| Vitamin B6, ug | 1866 | 2727 |
| Vitamin B7, ug | 55 | 132 |
| Vitamin B9-total folic acid, ug | 276 | 501 |
| Vitamin C ascorbic acid, mg | 135 | 210 |
