# Supplementary material for: A path to sustainable and healthy diets: modeling ovo-lacto-vegetarian food-based dietary guidelines
Source: Front Nutr. 2026 Jun 24;13:1754132. doi: 10.3389/fnut.2026.1754132 (PMC13341565; doi:10.3389/fnut.2026.1754132)
Supplement: Supplementary file 6 [file Table_6.docx]

Supplement 6: Total diet-related DALYs per food group for Austria, DALYs per 100 g of food i, and total DALYs of the optimized diet for Austria.

| Food group | Diet-related DALYs for Austria (GBD) (1) | Diet-related DALYs for Austria (Schw) (2) | $\boldsymbol{x}_{\boldsymbol{i}}^{\boldsymbol{Obs}}$ , observed mean intake in g/d (3) | $\boldsymbol{x}_{\boldsymbol{i}}^{\boldsymbol{TMREL}}$, TMREL | DALYs per 100 g of food group $\boldsymbol{i}$ (GBD) | DALYs per 100 g of food group $\boldsymbol{i}$  (Schw) | $\boldsymbol{x}_{\boldsymbol{i}}^{\boldsymbol{Opt}}$,optimized mean intake in g/d | DALYs_Opt_ (GBD) | DALYs_Opt_ (Schw) |
| --- | --- | --- | --- | --- | --- | --- | --- | --- | --- |
| Diet low in vegetables | 11.388 | 26.394 | 90 | 375 (2) | -3.989 | -9.245 | 375 | -14.958 | -34.668 |
| Diet low in fruit | 11.436 | 26.062 | 164 | 300 (1, 4, 5) | -8.384 | -19.107 | 131 | -10.983 | -25.030 |
| Diet low in legumes | 36.049 | 24.384 | 5 | 122 (2) | -30.680 | -20.752 | 12 | -3.682 | -2.490 |
| Diet low in nuts and seeds | 7.066 | 42.498 | 4 | 25 (1, 6) | -34.300 | -206.301 | 7 | -2.401 | -14.441 |
| Diet low in whole grains | 45.952 | 22.039 | 89 | 119 (2) | -153.687 | -73.709 | 17 | -26.127 | -12.531 |
| Diet high in refined grain |  | 1.291 | 87 | 0 (2) |  | 1.482 | 232 |  | 3.439 |
| Diet low in dairy |  | 11.563 | 150 | 355 (2) |  | -5.649 | 357 |  | -20.166 |
| Diet low in milk | 4.518 |  | 61 | 435 (2) | -1.206 |  | 325 | -3.921 |  |
| Diet high in eggs |  | 337 | 3 | 0 (2) |  | 9.912 | 38 |  | 3.766 |
| Diet low in fish |  | 22.683 | 16 | 131 (2) |  | -19.656 | 0 |  | 0 |
| Diet high in red meat | 39.197 | 7.828 | 27 | 0 (6) | 146.805 | 29.318 | 0 | 0 | 0 |
| Diet high in processed meat | 24.325 | 10.655 | 30 | 0 (2) | 80.279 | 35.165 | 0 | 0 | 0 |
| Diet high in sugar-sweetened beverages | 8.070 | 12.666 | 60 | 0 (2) | 13.473 | 21.145 | 0 | 0 | 0 |
|  |  |  |  |  |  |  |  |  |  |
| Total | 188.001 | 208.400 |  |  |  |  |  | **-62.072** | **-102.121** |

DALYs = Disability-Adjusted Life Years, GBD = Global Burden of Disease, Schw = Schwingshackl, TMREL = Theoretical minimum risk exposure level, DALYs_opt_= DALYs of the optimized diet, $x_{i}^{Obs}$ = observed intakes *x* of each food group *i,* $x_{i}^{Opt}$= optimized intakes *x* of each food group *i*

1. Afshin A, Sur PJ, Fay KA, Cornaby L, Ferrara G, Salama JS, et al. Health Effects of Dietary Risks in 195 Countries, 1990-2017: A Systematic Analysis for the Global Burden of Disease Study 2017. *The Lancet* (2019) 393(10184):1958-72. doi: 10.1016/S0140-6736(19)30041-8

2. Schwingshackl L, Knüppel S, Michels N, Schwedhelm C, Hoffmann G, Iqbal K, et al. Intake of 12 Food Groups and Disability-Adjusted Life Years from Coronary Heart Disease, Stroke, Type 2 Diabetes, and Colorectal Cancer in 16 European Countries. *European Journal of Epidemiology* (2019) 34(8):765-75. doi: 10.1007/s10654-019-00523-4

3. Elmadfa I, Hasenegger V, Wagner K, Putz P, Weidl N-M, Wottawa D, et al. Österreichischer Ernährungsbericht 2012. Wien: Bundesministerium für Gesundheit (2012). Available from: <https://ernaehrungsbericht2016.univie.ac.at/fileadmin/user_upload/dep_ernaehrung/forschung/ernaehrungsberichte/oesterr_ernaehrungsbericht_2012.pdf>

4. Micha R, Peñalvo JL, Cudhea F, Imamura F, Rehm CD, Mozaffarian D. Association between Dietary Factors and Mortality from Heart Disease, Stroke, and Type 2 Diabetes in the United States. *Jama* (2017) 317(9):912-24. Epub 2017/03/08. doi: 10.1001/jama.2017.0947

5. Gakidou E, Afshin A, Abajobir AA, Abate KH, Abbafati C, Abbas KM, et al. Global, Regional, and National Comparative Risk Assessment of 84 Behavioural, Environmental and Occupational, and Metabolic Risks or Clusters of Risks, 1990–2016: A Systematic Analysis for the Global Burden of Disease Study 2016. *The Lancet* (2017) 390(10100):1345-422. doi: <https://doi.org/10.1016/S0140-6736(17)32366-8>

6. Fadnes LT, Økland JM, Haaland Ø A, Johansson KA. Estimating Impact of Food Choices on Life Expectancy: A Modeling Study. *PLoS Med* (2022) 19(2):e1003889. Epub 2022/02/09. doi: 10.1371/journal.pmed.1003889
